# Model-guided gene circuit design for engineering genetically stable cell populations in diverse applications

**DOI:** 10.1098/rsif.2024.0602

**Published:** 2025-02-12

**Authors:** Kirill Sechkar, Harrison Steel

**Affiliations:** ^1^ Department of Engineering Science, University of Oxford, Parks Road, Oxford OX1 3PJ, UK

**Keywords:** gene circuit design, burden, resource competition, mathematical model, population, synthetic biology

## Abstract

Maintaining engineered cell populations’ genetic stability is a key challenge in synthetic biology. Synthetic genetic constructs compete with a host cell’s native genes for expression resources, burdening the cell and impairing its growth. This creates a selective pressure favouring mutations which alleviate this growth defect by removing synthetic gene expression. Non-functional mutants thus spread in cell populations, eventually making them lose engineered functions. Past work has attempted to limit mutation spread by coupling synthetic gene expression to survival. However, these approaches are highly context-dependent and must be tailor-made for each particular synthetic gene circuit to be retained. By contrast, we develop and analyse a biomolecular controller which depresses mutant cell growth independently of the mutated synthetic gene’s identity. Modelling shows how our design can be deployed alongside various synthetic circuits without any re-engineering of its genetic components, outperforming extant gene-specific mutation spread mitigation strategies. Our controller’s performance is evaluated using a novel simulation approach which leverages resource-aware cell modelling to directly link a circuit’s design parameters to its population-level behaviour. Our design’s adaptability promises to mitigate mutation spread in an expanded range of applications, while our analyses provide a blueprint for using resource-aware cell models in circuit design.

## 1. Introduction

Synthetic biology, which entails engineering living systems with useful functionalities by introducing new synthetic genes to cells, promises to tackle global challenges in medicine, industry and sustainability. However, synthetic biology is currently held back by several inherent challenges, such as non-modularity and evolvability of living systems, which make robust, predictable and durable performances difficult to achieve [1,2].

A major factor contributing to these issues is the finiteness of the pool of cellular resources (most importantly for bacteria, ribosomes) shared between synthetic genes introduced by engineers and the host cell’s own native genes. On the one hand, this complicates the design of so-called ‘circuits’ of synthetic genes regulating each other to sense, process and react to stimuli. Indeed, unlike electrical components, all gene circuit elements interact indirectly via the shared resource pool, which violates the engineering principle of modularity and complicates the prediction of a circuit’s performance based on the behaviours of its individual components observed in isolation [2,3]. Moreover, high synthetic gene expression may significantly deplete cellular resource pools, interfering with native gene expression and thus the cell’s growth and functioning. This burdens the host, which may have multiple downstream effects. The changed cellular context (e.g. cell-wide variations arising from growth rate changes) can impact circuits’ dynamics, making it even harder to predict their performance and design them accordingly [4,5]. Furthermore, in an engineered cell population, mutants with defunct circuitry may experience less burden and divide faster. This leads to ‘mutation spread’—growth of non-functional subpopulations that eventually outcompete and displace original cells. This evolutionary pressure to lose engineered functionalities significantly impairs biotechnologies’ productivity and durability [1].

To address resource finiteness challenges, synthetic biologists have developed mathematical gene expression models that incorporate resource competition dynamics [2], as well as the host cell’s growth regulation mechanisms and their interplay with synthetic gene expression [4]. This has enabled more reliable predictions of the genetic circuit performance despite the non-modularity and context-dependence of biological systems. Furthermore, insights provided by resource-aware model simulations and analytical derivations have facilitated the development of circuits rendering synthetic biology designs more modular and robust to resource competition [5].

To tackle resource competition’s population-level implications, several countermeasures to mutation spread have been proposed. Genes’ mutation probability can be lowered—although not eliminated—by optimizing synthetic genes’ DNA sequences and modifying the host’s genome [6]. Alternatively, synthetic gene expression burden can be reduced to make the engineered cell’s growth defect less of a competitive disadvantage. This can be done either permanently by giving circuit genes weaker promoters and ribosome-binding sequences (though at the cost of often-desired high expression levels), or only when synthetic protein synthesis excessively stresses the cell if stress-response promoters are used as negative feedback regulators [3]. In another strategy, engineered cells remain in a non-producing state (experiencing little burden), but a fraction of them in every generation is differentiated into a state with high synthetic gene expression [7]. Finally, to make a cell’s survival dependent on the presence of synthetic circuitry, a gene essential to cell growth is co-expressed with the synthetic genes of interest—potentially with these genes’ DNA sequences overlapping for even stronger coupling between their functioning. Loss of useful synthetic genes’ expression to mutation is thus accompanied by essential gene loss, making mutants unable to grow and outcompete engineered cells [6].

However, extant strategies for mitigating mutation spread often have restricted applications and are cumbersome to adapt for new scenarios. Many of the aforementioned methods limit the maximum achievable productivity of biotechnologies, either because they keep synthetic gene expression low by design (to reduce the burden and selective pressure against engineered cells) or due to confining the expression of genes of interest to just the subset of differentiated cells within the population. Such bounded synthetic capacity may be undesirable in applications like biomanufacturing, which require high product yields [3,6]. Furthermore, most methods require tailoring for each application. The sequence-specificity of codon optimization and essential gene overlapping mean that a given circuit’s DNA implementation must be redesigned with these strategies in mind. Likewise, the addition of a differentiation switch and co-expressed essential genes requires case-specific additional circuitry. Having to design and synthesize new DNA sequences for every new circuit of interest is time- and cost-intensive [8], which has limited the application of mutation spread mitigation methods in synthetic biology.

We propose ‘the Punisher’, a novel circuit for countering mutation spread. Our versatile design deprives mutant cells of a growth advantage, yet can be easily adapted for different applications without re-engineering its DNA sequences. This work extends the applications of resource-aware gene expression modelling techniques [5], previously used for mitigating the effects of resource competition on a single-cell level, to countering undesired population-wide burden phenomena. A coarse-grained cell model provides a holistic view of resource competition between synthetic and native genes, capturing its effects on cell growth. Based on this single-cell modelling, we define a novel engineered cell population model that directly links the circuit’s population-level performance to its parameters. Our analysis suggests scenarios in which the Punisher has advantages over the extant method of countering mutation spread by co-expressing synthetic and essential genes. Moreover, our modelling can help choose the Punisher’s design parameters, including those that can be adjusted without any genetic modifications, in order to re-use our circuit in novel applications.

## 2. Circuit design and analysis

### 2.1. Circuit description

**Figure 1.**
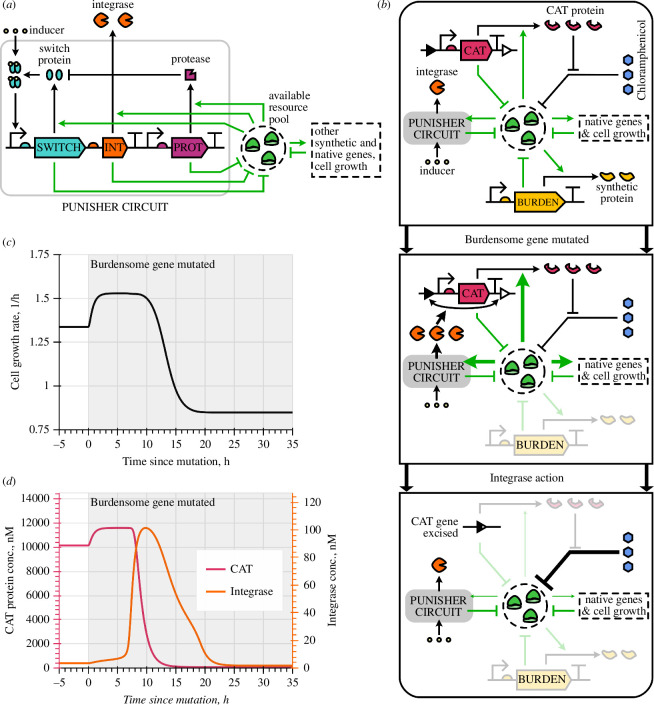
Basics of the Punisher's functioning. (*a*) The Punisher comprises a self-activating switch gene co-expressed with an integrase gene. The switch protein is degraded by a synthetic protease and, when bound by a chemical inducer molecule, acts as a transcription factor. All three proteins' expression depends on the availability of gene expression resources (ribosomes) in the cell. (*b*) The Punisher's response to synthetic gene expression loss (arrow boldness reflects the strength of interactions). When mutation of a burdensome synthetic gene frees up resources, increasing the mutant cell's growth rate, more integrase is produced by the Punisher. Consequently, the integrase excises the antibiotic resistance gene chloramphenicol acetyltransferase (CAT), so unhindered ribosome inactivation by the antibiotic chloramphenicol decreases the expression resources' availability and thus the mutant cell's growth rate. (*c,d*) Simulation of the Punisher's response to mutation of a single constitutive burdensome gene expressed in the same host cell.

Mutation spread occurs because cells in which synthetic genes mutate to become non-functional or non-expressed are rewarded by burden alleviation and the resultant growth advantage, which selects for such mutants and lets them eventually take over the population [1,6]. To counter this, our circuit, shown in figure 1*a*, replaces reward with punishment by detecting burden-alleviating mutations in its host *E. coli* cell and reducing cell growth in response. Responding directly to burden, regardless of which particular synthetic genes contribute to it, allows the Punisher to be easily re-used across different applications.

The Punisher’s detection component comprises the self-activating ‘switch’ gene, which encodes a transcription factor protein that, when bound by a chemical inducer molecule, promotes its own expression. Experiments show that such a protein’s concentration can converge to either a low- or a high-expression equilibrium depending on the burden experienced by the host cell [9,10]. When all synthetic genes in the cell are functional, competition for gene expression resources is high, so the switch protein’s concentration $p_{s}$ cannot reach a high-expression equilibrium. Upon synthetic gene expression loss, more resources become available for protein synthesis, so $p_{s}$ converges to the high-expression equilibrium, increasing several-fold. Since the timescale of a biomolecular species’ dynamics is primarily determined by the rate of its removal from the system [11], we speed up the Punisher’s response by having the switch protein be both diluted by cell division and degraded by a synthetic protease [12,13].

The punishment component is powered by an integrase protein co-expressed from the same operon with the switch gene. Once the integrase reaches a sufficiently high concentration, it excises the DNA sequence situated between its cognate sites, which flank a gene essential for cell growth. Hence, upon a mutation-induced rise in the switch’s and the integrase’s expression, the essential gene is excised from its plasmid or the cell’s genome [7,14], impairing host cell growth. Importantly, the integrase’s action is irreversible, so a ‘punished’ mutant cell remains slow-growing even if essential gene loss reduces resource availability, bringing the switch protein’s abundance back to its pre-detection level.

Our circuit’s operating principle is illustrated in figure 1*b* with the example of the cell hosting the Punisher alongside a single burdensome synthetic gene. Here, the essential gene excised by the integrase is chloramphenicol acetyltransferase (CAT), an enzyme which degrades the ribosome-inactivating antibiotic chloramphenicol [15]. If chloramphenicol is present in the culture medium, CAT gene loss thereby compromises translation, impairing cell growth. Initially, only a few integrase molecules are present in the cell, so CAT’s concentration is high and steady. Upon the burdensome gene’s mutation, the cell growth rate’s rise is followed by a sharp increase in the integrase’s abundance. Consequently, the CAT gene is excised and the cell growth rate slows down dramatically. Due to the excision’s irreversibility, the subsequent fall in the integrase’s concentration due to decreased ribosome availability does not recover the cell’s growth rate.

### 2.2. Modelling the circuit in the host cell context

Since the Punisher reacts to changes in gene expression resource availability and curbs the host cell’s growth rate in response, informative modelling of the circuit must incorporate resource competition dynamics between native and synthetic genes, as well as capture the mechanisms determining the host cell’s growth rate and physiological state [16,17]. We therefore employ a coarse-grained resource-aware cell model [5] which, besides synthetic circuitry, explicitly considers the expression of a cell’s native genes and their interactions and regulation. Including both synthetic and native genes’ contributions to resource demand, our model predicts the cell’s growth rate and resource allocation in a wide range of industrially relevant conditions, including when it is exposed to the ribosome-inhibiting antibiotic chloramphenicol, which significantly influences resource competition [4,16]. Simultaneously, coarse-graining the cell’s native genes by their function into just several classes keeps the model simple, allowing the derivation of analytical relations describing the Punisher’s behaviour in §2.3.

Our ordinary differential equation (ODE) system is thus based on the resource-aware cell model from [5], modified to capture effects of the synthetic protease and intracellular chloramphenicol as per electronic supplementary material, Note S1.3. Equations (2.1)–(2.6) describe host cell dynamics, whereas the behaviour of the synthetic gene set X is given by equations (2.7) and (2.8).


(2.1) $$\begin{array}{rl} {\dot{m}}_{a} & =c_{a}\alpha_{a}\lambda (\epsilon ,B)-(\beta_{a}+\lambda (\epsilon ,B))m_{a}, \end{array}\;$$

(2.2) $$\begin{array}{rl} {\dot{m}}_{r} & =F_{r}(t^{u},t^{c})\cdot c_{r}\alpha_{r}\lambda (\epsilon ,B)-(\beta_{r}+\lambda (\epsilon ,B))m_{r}, \end{array}\;$$

(2.3) $$\begin{array}{rl} {\dot{p}}_{a} & =\frac{\epsilon (t^{c})}{n_{a}}\cdot \frac{m_{a}/k_{a}}{D}R-\lambda (\epsilon ,B)\cdot p_{a}, \end{array}\;$$

(2.4) $$\begin{array}{rl} \dot{R} & =\frac{\epsilon (t^{c})}{n_{r}}\cdot \frac{m_{r}/k_{r}}{D}R-\lambda (\epsilon ,B)\cdot R, \end{array}\;$$

(2.5) $$\begin{array}{rl} \dot{t^{c}} & =\nu (t^{u},\sigma )\cdot p_{a}-\epsilon (t^{c})\cdot B-\lambda (\epsilon ,B)\cdot t^{c}, \end{array}\;$$

(2.6) $$\begin{array}{rl} \dot{t^{u}} & =\psi (t^{u},t^{c})\cdot \lambda (\epsilon ,B)-\nu (t^{u},\sigma )\cdot p_{a}+\epsilon (t^{c})\cdot B-\lambda (\epsilon ,B)\cdot t^{u}, \end{array}\;$$

(2.7) $$\begin{array}{rl} {\dot{m}}_{x_{l}} & =F_{x_{l}}(\cdot )c_{x_{l}}\alpha_{x_{l}}\lambda (\epsilon ,B)-(\beta_{x_{l}}+\lambda (\epsilon ,B))m_{x_{l}}\;\;\;\;\;\;\;\;\;\;\;\;\;\text{for all }x_{l}\in X, \end{array}\;$$

(2.8) $$\begin{array}{rl} {\dot{p}}_{x_{l}} & =\frac{\epsilon (t^{c})}{n_{x_{l}}}\cdot \frac{m_{x_{l}}/k_{x_{l}}}{D}R-(\delta_{x_{l}}p_{prot}+\lambda (\epsilon ,B))p_{x_{l}}\;\;\;\;\;\;\;\;\;\;\;\text{for all }x_{l}\in X. \end{array}$$

Gene expression resource allocation and growth regulation in *E. coli* are primarily determined by tRNA charging in the cell [16,18]. Therefore, our model includes concentrations of aminoacyl-tRNAs $t^{c}$ and uncharged tRNAs $t^{u}$ and considers two classes of the cell’s native genes with opposite effects on tRNA levels. Metabolic genes (a) aminoacylate tRNAs using nutrients from the culture medium, whose quality is captured by the factor σ. Meanwhile, ribosomal genes (r) are responsible for protein synthesis, which consumes charged tRNAs. The a and r gene classes are each treated as a single lumped gene, whose mRNA concentrations are $m_{a}$ and $m_{r}$, respectively, and whose protein concentrations are respectively $p_{a}$ and R. Out of R nM of ribosomes in the cell, B nM are translating. Both for native and synthetic genes, $c_{j}$ is the gene j’s DNA concentration in the cell, $\alpha_{j}$ is its promoter strength, $n_{j}$ is its length in amino acids, $\beta_{j}$ is the mRNA degradation rate and $k_{j}$ is the mRNA-ribosome dissociation constant, reflective of a gene’s ribosome-binding sequence (RBS) strength. Synthetic gene ODEs also include the (possibly zero) rate $\delta_{x_{l}}$ at which the Punisher’s protease (present in concentration $p_{prot}$) degrades them, as well as the transcription regulation function $0\leq F_{x_{l}}(\cdot )\leq 1$ whose form and arguments are gene-specific. The functions λ(ϵ,B), $\epsilon (t^{c})$, $F_{r}(t^{u},t^{c})$, $\psi (t^{u},t^{c})$ and $\nu (t^{u},\sigma )$ are given in electronic supplementary material, table S2, and represent the cell growth rate, the translation elongation rate, the ribosomal genes’ transcription regulation function and tRNA synthesis and aminoacylation rates, respectively.

The ‘ribosomal competition denominator’ D capturing the cell’s translational resource availability is defined in equations (2.9) and (2.10) which, besides the already-explained variables, include the mass fraction of housekeeping (i.e. not metabolic or ribosomal) native proteins in the cell ϕq, assumed constant [5,15], and the intracellular concentration h of chloramphenicol, which binds and inactivates ribosomes with a dissociation constant $K_{D}$.


(2.9) $$\begin{array}{c} D=\frac{K_{D}+h}{K_{D}}\cdot \left(1+\frac{\sum_{j\in \{a,r\}\cup X}m_{j}/k_{j}-\frac{K_{D}+h}{K_{D}}\cdot \frac{{\bar{\phi }}_{q}\Delta }{\epsilon R}}{1-{\bar{\phi }}_{q}\left(1-\frac{K_{D}+h}{K_{D}}\cdot \frac{\Delta }{\epsilon R}\right)}\right), \end{array}$$

(2.10) $$\begin{array}{r} \text{where }\Delta =p_{prot}\sum_{x_{l}\in X}n_{x_{l}}\delta_{x_{l}}p_{x_{l}} \end{array}.$$

The synthetic gene set X includes synthetic circuitry whose mutation the Punisher aims to penalize—the ODEs characterizing different set-ups considered in this study are provided in electronic supplementary material, Note S1.5. Moreover, X includes the Punisher’s switch, integrase, protease and CAT genes (X⊇{s, i, prot, cat}) described by equations (2.11)–(2.19), where I is the share of switch proteins bound by chemical inducer molecules, $\eta_{s}$ and $K_{s}$ are the cooperativity and the dissociation constant for the switch protein’s binding to the switch gene’s DNA, and $F_{s_{b}}$ is the switch gene promoter’s baseline activity without transcriptional activation.


(2.11) $$\begin{array}{rl} {\dot{m}}_{s} & =F_{s}(p_{s},I)\cdot c_{s}\alpha_{s}\lambda (\epsilon ,B)-(\beta_{s}+\lambda (\epsilon ,B))m_{s}, \end{array}$$

(2.12) $$\begin{array}{rl} {\dot{p}}_{s} & =\frac{\epsilon (t^{c})}{n_{s}}\cdot \frac{m_{s}/k_{s}}{D}R-(\delta_{s}p_{prot}+\lambda (\epsilon ,B))p_{s}, \end{array}\;$$

(2.13) $$\;\begin{array}{rl} m_{i} & \equiv m_{s}\cdot \frac{n_{i}}{n_{s}}\;\text{due to co-expression}\;(\text{see ESM, Note S1.2}), \end{array}$$

(2.14) $$\begin{array}{rl} {\dot{p}}_{i} & =\frac{\epsilon (t^{c})}{n_{i}}\cdot \frac{m_{i}/k_{i}}{D}R-(\delta_{i}p_{prot}+\lambda (\epsilon ,B))p_{i}, \end{array}$$

(2.15) $$\begin{array}{rl} {\dot{m}}_{prot} & =c_{prot}\alpha_{prot}\lambda (\epsilon ,B)-(\beta_{prot}+\lambda (\epsilon ,B))m_{prot}, \end{array}$$

(2.16) $$\;\begin{array}{rl} {\dot{p}}_{prot} & =\frac{\epsilon (t^{c})}{n_{prot}}\cdot \frac{m_{prot}/k_{prot}}{D}R-(\delta_{prot}p_{prot}+\lambda (\epsilon ,B))\cdot p_{prot}, \end{array}$$

(2.17) $$\begin{array}{rl} {\dot{m}}_{cat} & =c_{cat}\alpha_{cat}\lambda (\epsilon ,B)-(\beta_{cat}+\lambda (\epsilon ,B))m_{cat}, \end{array}\;$$

(2.18) $$\;\begin{array}{rl} {\dot{p}}_{cat} & =\frac{\epsilon (t^{c})}{n_{cat}}\cdot \frac{m_{cat}/k_{cat}}{D}R-(\delta_{cat}p_{prot}+\lambda (\epsilon ,B))p_{cat}, \end{array}$$

(2.19) $$\begin{array}{rl} F_{s}(p_{s},I) & =F_{s_{b}}+(1-F_{s_{b}})\cdot \frac{(Ip_{s}{)}^{\eta_{s}}}{(Ip_{s}{)}^{\eta_{s}}+K_{s}^{\eta_{s}}}. \end{array}\;$$

While all other genes’ concentrations remain constant, CAT gene DNA can be excised by the integrase in a reversible strand exchange reaction followed by an irreversible conformation change. We model this using equations (2.20) and (2.21) derived in electronic supplementary material, Note S1.4, based on an experimentally parametrized serine integrase action model [14,19]. Here, $c_{cat}$ and $c_{LRi}$ are concentrations of the CAT gene DNA before and after strand exchange (the former being the functional gene copy number), $K_{bI}$ is the integrase-DNA dissociation constant, $k_{sx}^{+}$ and $k_{sx}^{-}$ are the forward and backward strand exchange rates and $k^{conf}$ is the conformation change rate.


(2.20) $$\begin{array}{rl} {\dot{c}}_{cat} & =-k_{sx}^{+}\frac{p_{i}^{4}}{K_{bI}^{4}+p_{i}^{4}}c_{cat}+k_{sx}^{-}c_{LRi}, \end{array}\;$$

(2.21) $$\;\begin{array}{rl} {\dot{c}}_{LRi} & =k_{sx}^{+}\frac{p_{i}^{4}}{K_{bI}^{4}+p_{i}^{4}}c_{cat}-k_{sx}^{-}c_{LRi}-(k^{conf}+\lambda )c_{LRi}. \end{array}$$

Capturing the rate at which CAT binds and degrades chloramphenicol by the affinity constant $K_{C}$, we define equation (2.22) for chloramphenicol’s intracellular concentration, where κ is the rate of chloramphenicol’s diffusion through the cell membrane [15,20].


(2.22) $$\dot{h}=\kappa (h_{ext}-h)-\frac{hp_{cat}}{K_{C}}-\lambda (\epsilon ,B)\cdot h.$$

We use these ODEs to simulate the case of the cell hosting a single constitutive synthetic gene alongside the Punisher, described in §2.1 and electronic supplementary material, Note S1.5.1. The obtained trajectory in figure 1*c,d* matches our expectations for the Punisher’s performance. This behaviour is reproduced by stochastic cell model trajectories, simulated according to the hybrid tau-leaping method described in [5] (electronic supplementary material, Note S2.1). The code for these and all other simulations, available at [21], was implemented in Python 3.12 using the JAX 0.4.23 package to enable efficient parallelized computation [22,23].

### 2.3. Switching threshold identification and tuning

As described in §2.1, the Punisher detects synthetic gene mutations if they reduce the burden experienced by the host cell below the threshold value at which the switch gene transitions from a low-expression to a high-expression equilibrium. Thus, predicting whether the Punisher will function correctly requires understanding what determines its switching threshold and how to adjust it. Moreover, although multiple different synthetic circuits may burden the cell to a similar extent (allowing to use the same implementation of the Punisher with either of them), synthetic gene expression burden may vary depending on the circuit’s architecture and the cell culture conditions. Therefore, deploying the Punisher in new scenarios, where synthetic gene mutations alleviate burden to a different extent, also requires guidance on adjusting the Punisher’s switching threshold. Such design insights are enabled by our coarse-grained cell model’s simplicity and amenability to analytical derivations.

Electronic supplementary material, Note S3, shows that, with several realistic simplifying assumptions, our coarse-grained resource-aware cell model allows to quantify the steady-state burden of expressing a native or synthetic gene j as


(2.23) $$\xi_{j}=\frac{{\bar{F}}_{j}\alpha_{j}c_{j}}{{\bar{k}}_{j}},$$

where Fj is the steady-state value of its transcription regulation function. The total gene expression burden sensed by the Punisher is the sum of these values over all genes except for the Punisher’s switch and integrase genes themselves,


(2.24) $$\Xi =\sum_{j\in \{a,r\}\cup X\setminus \{s,i\}}\xi_{j}.$$

Solving the model at steady state retrieves the value of $F_{s}$ required to achieve a given steady-state switch protein concentration $p_{s}$ for a given burden. This ${\bar{F}}_{s}^{req}$ value is defined in equations (2.25) and (2.26) with ϵ and h standing for the steady-state translation elongation rate and intracellular chloramphenicol concentration, M being the host cell’s total protein mass, and $\xi_{s}^{max}$ and $\xi_{i}^{max}$ representing the maximum (i.e. calculated for $F_{s}=F_{i}=1$) burden of expressing the switch and the integrase proteins.


(2.25) $$\begin{array}{c} {\bar{F}}_{s}^{req}(p_{s},\Xi )=p_{s}(1+\chi )\cdot \frac{\Xi }{\xi_{s}^{max}+\xi_{i}^{max}}\cdot {\left(\frac{M(1-{\bar{\phi }}_{q})}{n_{s}}\cdot \frac{\xi_{s}^{max}}{\xi_{s}^{max}+\xi_{i}^{max}}-p_{s}(1+\chi )\right)}^{-1}, \end{array}$$

(2.26) $$\begin{array}{c} \text{where }\chi =\frac{K_{D}+\bar{h}}{\bar{h}}\cdot \frac{\delta_{s}Mn_{r}\xi_{prot}}{\epsilon n_{prot}\xi_{r}}. \end{array}$$

**Figure 2.**
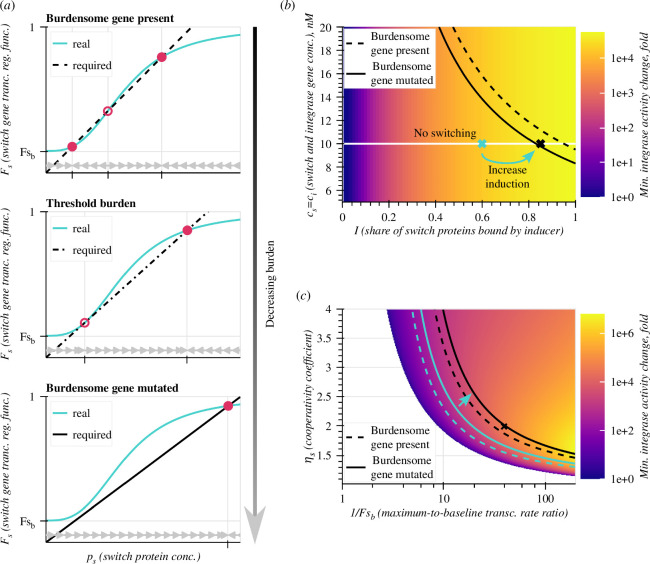
Emergence, identification and tuning of the Punisher's switching threshold. (*a*) Required (Fsreq) and real (${\bar{F}}_{s}^{real}$) values of the switch gene's transcription regulation function calculated from the switch protein's concentration $p_{s}$ for all synthetic genes being functional (top), at the threshold burden $\hat{\Xi }$ (middle), and with synthetic gene expression lost (bottom). Circles mark the system's fixed points—filled for stable, empty for unstable. Grey arrows mark the direction of convergence of $p_{s}$. (*b*) Heatmap of the minimum fold-change in the integrase's DNA-cutting activity as a function of the switch and integrase genes' DNA concentration $c_{s}=c_{i}$ and the share I of switch proteins bound by the inducer. Dashed and solid black lines respectively represent parameter combinations for which the switching threshold $\hat{\Xi }$ is equal to burden with and without the expression of the single constitutive synthetic gene. The Punisher functions correctly for all parameter combinations in the acceptable region between these lines. The black cross represents the parameter combination used in simulations for figure 1. If initially the Punisher's parameters do not make it switch on upon synthetic gene expression loss (e.g. blue cross at I=0.6), the inducer's concentration in the medium can be tuned to achieve the desired behaviour (blue arrow). (*c*) Heatmap of the minimum fold-change in the integrase's activity as a function of the switch gene's cooperativity coefficient $\eta_{s}$ and the maximum-to-baseline promoter activity ratio $1/F_{s_{b}}$. In the blank region, switching does not occur at any Ξ value. Likewise to (*b*), black lines denote parameter combinations for which $\hat{\Xi }$ is equal to burden with and without synthetic burdensome gene expression. The acceptable parameter region is flanked by them. Meanwhile, blue lines mark the acceptable parameter region for I=0.6, which can be shifted by increasing I (blue arrow) to cover the $F_{s_{b}}$ and $\eta_{s}$ value combination used in figure 1 (black cross).

However, the switch gene’s transcription regulation function’s actual value is given by a Hill function in equation (2.19). The switch protein concentration is therefore in steady state if and only if


(2.27) $${\bar{F}}_{s}^{req}(p_{s},\Xi )={\bar{F}}_{s}^{real}(p_{s})\;\;\;\Leftrightarrow \;\;\;{\bar{F}}_{s}^{req}(p_{s},\Xi )=F_{s_{b}}+(1-F_{s_{b}})\cdot \frac{(Ip_{s}{)}^{\eta_{s}}}{(Ip_{s}{)}^{\eta_{s}}+K_{s}^{\eta_{s}}},$$

i.e. the real transcription regulation function’s value is equal to the value required to achieve equilibrium. Graphically, this can be understood as the blue and the black curves intersecting in figure 2*a*. The plot also reveals the fixed points’ stability: the switch protein’s concentration decreases when the actual transcription rate is below that required to keep $p_{s}$ steady, and increases otherwise.

Equation (2.25) indicates that Ξ defines the black line’s gradient in figure 2*a*. Hence, for large gene expression burden this line rises steeply and crosses Fsreal at low $p_{s}$, producing a stable equilibrium (figure 2*a*, top). As Ξ is reduced, the curve’s slope becomes gentler, until it touches Fsreal from below, producing a bifurcation with a saddle node at low $p_{s}$ and a stable fixed point to its right (figure 2*a*, middle) [10]. If burden further decreases below this threshold, a single high-expression equilibrium remains (figure 2*a*, bottom). Crucially, since the switch gene and the integrase are co-regulated, the low-expression equilibrium stands for low integrase abundance and CAT gene excision rate ($p_{i}\ll K_{bI}$), whereas the high-expression fixed point corresponds to high integrase concentration and thus high essential gene excision rate.

Therefore, the Punisher penalizes synthetic gene mutation when it brings the burden Ξ below the bifurcation threshold $\hat{\Xi }$. Retrieving this threshold value from the Punisher’s design parameters (see electronic supplementary material, Note S3.4) and comparing it with burden before and after synthetic gene mutation allows to predict whether the Punisher will become activated in a mutant cell. Moreover, a lower bound on the change in integrase activity upon the Punisher’s triggering can be found according to electronic supplementary material, Note S3.5, revealing how the Punisher’s design parameters define its performance. For instance, figure 2*b* shows its dependence on the switch and integrase genes’ copy number and the inducer’s concentration in the culture medium. Meanwhile, figure 2*c* depicts the effects of the cooperativity coefficient $\eta_{s}$ and the baseline promoter activity $F_{s_{b}}$, which are key determinants of a self-activating gene’s equilibria [24].

The dependence of $\hat{\Xi }$ on I is particularly significant. While all other parameters are determined by the synthetic DNA sequence during construct design, the inducer’s concentration can be easily tuned by adjusting the chemical inducer’s concentration in the culture medium. Graphically, it stands for moving along the white line in figure 2*b* to position $\hat{\Xi }$ between the pre- and post-mutation burden levels. In plots for other parameters, such as figure 2*c*, changing the chemical induction is equivalent to moving the acceptable parameter region to cover a given parameter combination. Therefore, without any re-engineering of its genetic components, the same DNA implementation of the Punisher can be re-used in different conditions and with sundry synthetic gene circuits of varying burdensomeness simply by adding different inducer amounts to the medium.

## 3. Example application

### 3.1. Performance simulation

**Figure 3.**
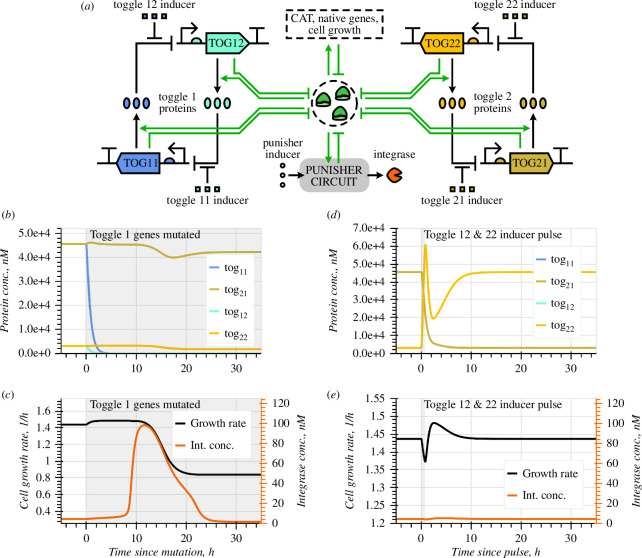
Using the Punisher with two synthetic toggle switch circuits. (*a*) Schematic of two toggle switches being expressed in the same cell as the Punisher. (*b,c*) Simulation of the Punisher's response to loss of expression of the first toggle's both genes. (*d,e*) Simulation of the Punisher's response to two toggles being flipped by a transient inducer concentration pulse.

We now simulate the Punisher’s deployment alongside more complex synthetic gene circuitry than that shown in figure 1. Namely, we consider two synthetic toggle switches (figure 3*a*), described with ODEs in electronic supplementary material, Note S1.5.2. A toggle switch comprises two genes that repress each other’s expression; this repression’s strength can be modulated by adding chemical inducers to the medium. Therefore, for certain combinations of design parameters and environmental conditions, a toggle circuit can exhibit bistability, since either of the toggle’s two genes can be highly expressed whilst repressing the other gene’s expression. Pulses of inducer concentration can ‘flip’ the toggle from one equilibrium state to the other, where it stays until the next flipping [25]. Several toggle switches may be required in the same cell if it needs to simultaneously ‘remember’ several different inducer pulses.

Importantly, loss of expression of a single synthetic gene in a network does not always alleviate burden [1]. Namely, mutating one gene in a toggle switch means that the other gene is no longer repressed and is thus expressed even more actively, increasing burden and slowing down cell growth (electronic supplementary material, Note S2.2). Therefore, single-gene mutants have a growth disadvantage compared with original engineered cells, presenting no risk of outcompeting them in the population. Losing both genes of a toggle switch, conversely, may significantly increase resource availability. In line with our derivations in §2.3, the inducer level should thus be changed to set I=0.87 and position the Punisher’s switching threshold between the burden of expressing both toggle switches and that of expressing one toggle. As revealed by figure 3*b*,*c*, the Punisher then detects the loss of a toggle and reduces cell growth in response.

When a synthetic circuit is out of steady state, the burden caused by it can vary over time. For instance, when a toggle switch is flipped, the overall expression of its genes may momentarily dip. Simultaneously flipping both toggles can temporarily reduce burden almost as much as mutating one toggle switch. Nonetheless, the Punisher can reject (i.e. not respond to) such transient disturbances (figure 3*d*,*e*). Provided that burden alleviation is sufficiently short-lived, the Punisher still may not leave the low-expression equilibrium’s basin of attraction by the time the burden returns to its original high value; therefore, the Punisher goes back to the off-state without triggering essential gene excision. As shown in electronic supplementary material, Note S3.6, knowing the expected duration of disturbance to be filtered out, the Punisher’s switching timescale can be adjusted accordingly via methods which require minimal or no DNA editing [26]. Besides rejecting perturbations associated with gene circuits’ dynamic behaviour, this feature of the Punisher can also render it robust to cell cycle-associated fluctuations (electronic supplementary material, Notes S2.6−2.7) [11,27,28].

### 3.2. Comparison with alternative mutation spread mitigation strategies

When cell populations need to retain relatively complex circuits, the burden-sensing nature of the Punisher’s response can make it advantageous over extant mutation spread mitigation strategies. An instance of this is the task of penalizing the mutations of two toggle switches, fulfilled using the Punisher in §3.1. Here, we compare our design’s performance in this case with that of the co-expression method discussed in §1 and [6], where the synthetic gene to be retained by the cell population is expressed together with a gene essential for growth, so mutating the former also disables the latter.

**Figure 4.**
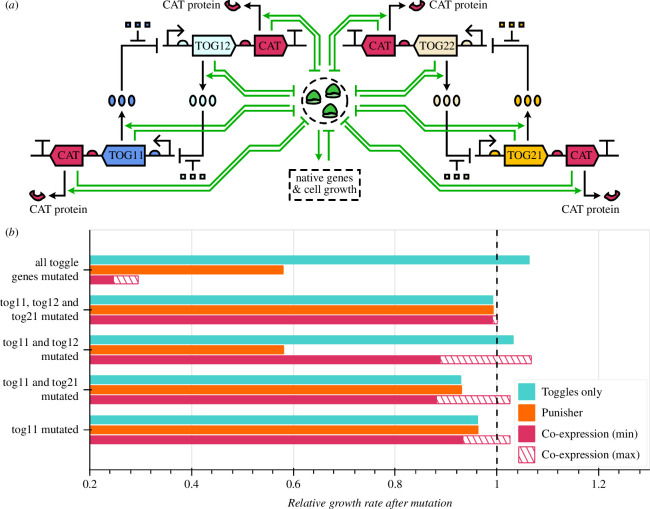
Comparing the Punisher's and essential gene co-expression's ability to penalize mutations of two synthetic toggle switches. (*a*) Schematic of two toggle switches with all genes co-expressed with essential CAT gene copies. (*b*) The host cell's steady-state growth rates (obtained by simulating the system for 50h) with certain genes mutated relative to its growth rate with all synthetic circuitry fully functional. The two toggle switches were assumed to be present in the cell without any mutation-penalizing circuitry, alongside the Punisher as shown in figure 3, or co-expressed with the CAT gene as per (*a*). For the latter, we consider CAT mRNA-ribosome association rates $0.24<k_{cat_{yz}}^{+}<60$ nM^−1^ h^−1^ [4,29,30], showing the minimum and maximum relative growth rates in this RBS strength range.

We simulate this scenario as depicted in figure 4*a* and described with ODEs in electronic supplementary material, Note S1.5.3. Here, the cell hosts two synthetic toggles whose genes (for symmetry, all four of them) are co-transcribed in the same operon with the antibiotic resistance gene CAT. Despite being translated from the same mRNA, essential and toggle gene expression rates may differ due to distinctions in post-transcriptional behaviour, which we capture by considering a wide range of possible CAT gene RBS strengths [4,29,30]. Figure 4*b* demonstrates that for most synthetic gene mutation combinations the Punisher slows down mutant cells’ growth comparably to the best possible co-expression set-up.

More significantly, in some parameter regimes CAT gene co-expression in fact promotes the growth of cells with undesirable mutations, which would otherwise be unable to take over the cell population. This is because the co-expression method operates not with the burden which slows down cell growth, but rather with mutations themselves, whose influence on growth rates may be less straightforward [1,6]. For instance, mutating one gene in a toggle upregulates its counterpart, formerly repressed by it (electronic supplementary material, Note S2.3). This can increase synthetic protein expression levels above its original values, elevating the expression burden—which in this case is desirable as it selects against mutant cells. However, if this newly de-repressed gene is co-expressed with an essential gene, the benefit to cell viability from increased essential protein levels can counteract this useful additional burden, potentially even producing mutant cells that grow faster than their unmutated progenitors. By contrast, the Punisher only reacts to synthetic gene expression loss when it does reduce burden and increase cell growth rates, removing the possibility of responses that actively (and undesirably) promote mutation spread.

Engineering essential gene co-expressions can also be more cumbersome than deploying the Punisher. First, a circuit may require multiple co-expressions (e.g. four of them in our case), implementing which in DNA is time- and cost-intensive [8]. To apply the co-expression method to another circuit, this DNA engineering step would have to be repeated anew. Second, only certain parameter regions (e.g. RBS strengths $0.66<k_{cat_{yz}}^{+}<1.63$ nM^−1^ h^−1^ in our case) yield co-expression set-ups which do not risk inadvertently promoting mutation spread. Meanwhile, parameter tuning for this method can be challenging. Adjusting RBS strengths requires DNA editing, while the design space for ribosome-binding sequences may be restricted if the essential gene is not merely co-expressed, but actually overlaps with a synthetic gene’s sequence for stronger protection against mutations [6]. CAT genes’ transcription, the most commonly tuned expression step in synthetic biology, is governed by the toggles’ promoters and thus is not easily adjusted. Less trivial post-transcriptional regulation circuitry, which can be complicated to implement, may therefore be necessary to enable a functional essential gene co-expression set-up.

Conversely, the same DNA implementation of the Punisher can be re-used in different applications. Although our design, too, is only effective within a certain parameter region (see figure 2), the switching threshold can be adjusted without costly genetic interventions by changing the concentration of its inducer in the medium as shown in §2.

## 4. Population-scale simulations

### 4.1. Population model definition

Previous sections demonstrate that the Punisher can detect synthetic gene mutation-induced changes in the burden experienced by a single cell, curbing its growth in response. However, the Punisher’s ultimate purpose is to slow down the outcompetition of engineered cells by mutants in a population, so *in silico* evaluation of our design’s performance ultimately requires population-level modelling. We therefore propose a model of a population of cells in a bioreactor, defined for the case of the Punisher favouring the retention of a single synthetic burdensome gene as described in §2.

Given the very high number of cells in a typical bioreactor [1], agent-based approaches capturing each individual cell’s behaviour with a separate model and explicitly considering all intercellular interactions [31] are computationally intractable. Instead, population-scale models, frequently used in evolutionary and synthetic biology [7], can classify cells as members of subpopulations according to their genetic state—i.e. which synthetic genes remain unmutated and functional (meanwhile, electronic supplementary material, Note S4.2, shows that native gene mutations conferring cells with chloramphenicol resistance are unlikely to significantly affect population dynamics [32]). Each cell type’s abundance is a variable modelled with an ODE. The combination of this approach with resource-aware cell modelling was pioneered by Ingram and Stan [1]. However, their model assumed identical internal state dynamics (captured by an ODE cell model) for every cell in a given genetic state. This is unsuitable for modelling genetic circuits like the Punisher, which takes time to become activated upon synthetic gene mutation, hence the state of a recently mutated cell’s circuitry being different to that of a cell mutated long ago.

**Figure 5.**
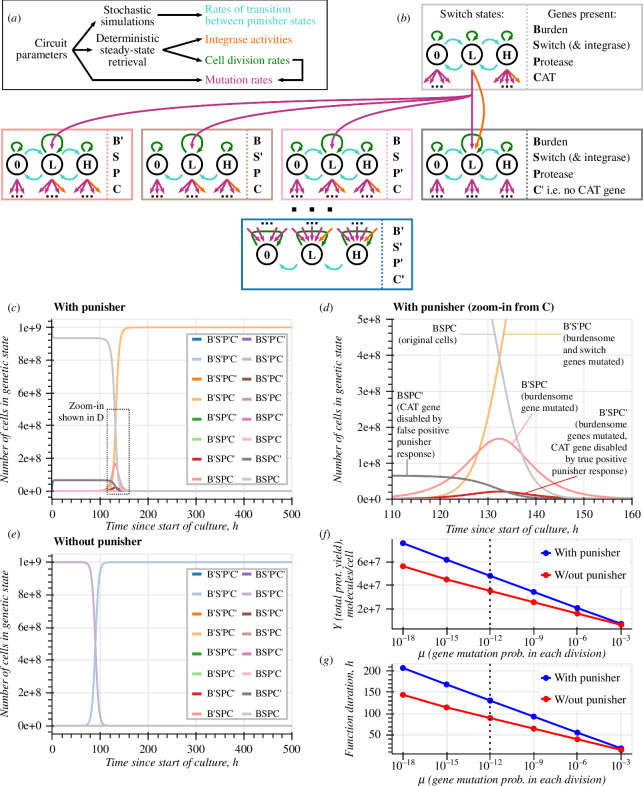
Modelling a population of cells hosting a synthetic burdensome gene and the Punisher. (*a,b*) The population is split into subpopulations of cells based on their genetic state (i.e. which synthetic genes are still present or have been mutated) and state of the Punisher's switch gene (zero, low or high expression). Transition rates between the Punisher's states are found by stochastic single-cell simulations. The cell changes its genetic state either due to mutations during cell divisions or due to integrase action, the rates of which are found by deterministic single-cell simulations. Each subpopulation replenishes itself by cell division, whose rate is also found deterministically. (*c–e*) Simulations of the population model with and without the Punisher. Every synthetic gene is assumed to mutate with a probability of $\mu =10^{-12}$ with every cell division. (*f–g*) Total protein yield per cell and function duration of engineered cell populations with and without the Punisher for different synthetic gene mutation probabilities per cell division. The dotted line represents $\mu =10^{-12}$ used in (*c–e*).

We thus further subdivide genetically identical cells according to the Punisher’s state in each cell and characterize the rates of switching between circuit states by simulating our resource-aware single-cell model (figure 5*a*). The resultant model is depicted in figure 5*b* and described and parametrized in electronic supplementary material, Note S5. The cell population is split into $2^{4}=16$ genetic states based on the functionality of: the burdensome gene to be retained (B); the switch and integrase genes (S, treated as one gene due to being co-expressed); the synthetic protease gene (P); and the CAT gene (C). If a given gene is mutated, the genetic state’s index has a ′ mark after the corresponding letter. Each genetic state can have three possible states of the Punisher—the high-expression equilibrium (H), the low-expression equilibrium (L) or else no integrase and switch proteins present (0), e.g. when the Punisher has mutated and all its previously synthesized proteins have been removed from the cell. If some equilibrium does not actually exist in a given genetic state (like the low-expression equilibrium when the burdensome gene is non-functional), the corresponding cell state’s properties are assumed identical to those of its closest unmutated progenitor according to electronic supplementary material, table S7. We thus have 16×3=48 variables in total, each representing the abundance of cells with a given genetic state and a given state of the Punisher. The index $j=B^{\prime }SPC:H$, for example, stands for cells with the Punisher in the high-expression equilibrium and only the synthetic burdensome gene B being non-functional.

Each cell in state j divides at a rate $\lambda_{j}$ to produce two daughter cells, usually in the same state—however, with probability μ a still-functional gene may mutate, making the daughter cells contribute to another genetic state’s cell count. A genetic state transition can also be caused by the CAT gene’s excision at a rate dependent on the integrase’s concentration (i.e. on the Punisher’s state). Genetic changes do not directly affect the Punisher; instead, the Punisher’s transition rates are fixed but dependent on the cell’s genetic state. We determine them by simulating our resource-aware single-cell model stochastically [33]. This captures both ‘true positive’ detection of mutations predicted by ODE simulations and ‘false positive’ activations of the Punisher due to the stochasticity of gene expression, as well as accounting for the effects of stochasticity on the timescale of the self-activating switch gene’s expression dynamics [24].

In summary, the evolution of cell counts in the bioreactor is given by


(4.1) $$\dot{x\;x}=(D\;D+A\;A+T\;T-L(x\;x,d\;d))x\;x,$$

where x is the 48-dimensional vector of cell counts by the state and d is the vector of corresponding cell division rates.

The matrix DD contains rates of different states’ cell counts changing due to cell division (which includes the possibility of mutations). The matrix TT captures transitions between the states of the Punisher, whereas A represents rates of the cells’ genetic state changes due to integrase action. Definitions for d, D, A and T are provided in electronic supplementary material, Note S5. Finally, if the bioreactor is a turbidostat keeping the overall cell abundance constant [15], it dilutes all cells at the rate


(4.2) $$L(x\;x,d\;d)=\frac{x\;x\cdot d\;d}{sum(x\;x)}.$$

### 4.2. Population simulation results

To gauge the Punisher’s population-level performance, we integrated equation (4.1) over time, starting at the initial condition where all cells in the bioreactor had all synthetic genes unmutated and the Punisher in a low-expression equilibrium, i.e.


(4.3) $$\begin{array}{r} x\;x_{j}(0)=\left\{ \begin{array}{ll} 10^{9} & \text{ if }j=BSPC:L\\ 0 & \text{ otherwise} \end{array}\right. \end{array}.$$

For comparison, we simulated a population of cells lacking the Punisher’s switch, integrase and protease (hence the zero switch and integrase protein level), but still hosting the burdensome and CAT genes. This stands for the initial condition


(4.4) $$\begin{array}{r} x\;x_{j}(0)=\left\{ \begin{array}{ll} 10^{9} & \text{ if }j=BS^{\prime }P^{\prime }C:0\\ 0 & \text{ otherwise} \end{array}\right. \end{array}.$$

Representative simulated trajectories for cell populations with and without the Punisher, plotted in figure 5*c,d,e*, qualitatively confirm that our design allows to prolong the prevalence of cells with a functional burdensome gene. A quantitative measure of an engineered cell population’s productivity is its rate of burdensome protein synthesis per cell Θ, defined in equation (4.5), where $p_{b}(j)$ is the burdensome protein content of a cells in state j, found by simulating our ODE single-cell model [7].


(4.5) $$\Theta =\frac{\sum_{j}x\;x_{j}\cdot d\;d_{j}\cdot p_{b}(j)}{\sum_{j}x\;x_{j}}.$$

To gauge the cells’ total productivity over time, Θ can be integrated over the culture duration $t_{cult}=500$ to find the total burdensome protein yield per cell Y. Moreover, Θ can be tracked over time to find a population’s function duration τ, which we define as the time for which H remains above 50% of its maximum value. Plotting these metrics in figure 5*f*,g demonstrates that the Punisher increases cell population productivity’s robustness to mutations roughly 1.5-fold over a wide range of gene mutation rates.

The Punisher’s observed beneficial effect on the engineered population’s function duration is explained by the cells having to accumulate two mutations, instead of just one, in order to gain a substantial growth advantage. As shown in figure 5*d*, cells with just the burdensome gene mutated (state $B^{\prime }SPC$) multiply very slowly, becoming noticeable only at t≈100 h. Indeed, the Punisher detects the reduced burden in them and disables their CAT gene. The resultant $B^{\prime }SPC^{\prime }$ cells divide very slowly, being rapidly diluted out of the bioreactor. The rapid displacement of original engineered cells therefore only becomes possible when a (rare) $B^{\prime }SPC$ mutant also mutates the Punisher’s switch and integrase genes to escape penalization.

In practice, delaying mutation spread’s onset with the Punisher may prove even more beneficial for the cell population’s productivity than predicted, which can be understood through the lens of the clonal interference phenomenon [34]. Besides synthetic gene mutations, cells in a bioreactor may undergo native gene mutations with a growth advantage which, while not triggering the Punisher, may exceed the benefit gained from synthetic gene expression loss (see electronic supplementary material, Note S4.1). Therefore, the Punisher’s extension of the time throughout which original engineered cells predominate in the population increases the chance of such advantageous native gene mutations first arising in the cells with fully functional synthetic circuitry. This may allow them to outgrow undesirable cells with a mutated synthetic burdensome gene.

## 5. Discussion

In summary, we have leveraged known resource competition phenomena to design a novel versatile biomolecular controller that counters mutation spread in engineered cell populations. Simulations show that it can successfully disable cell growth upon burden-reducing mutations of different synthetic circuits’ genes, hindering the takeover of engineered cell populations by mutants (§§2.1, 3.1 and 4). Importantly, the same DNA implementation of the Punisher can be re-used across various applications simply by adjusting the culture medium’s chemical inducer content (§2), which avoids costly DNA redesign and synthesis steps characteristic of extant mutation spread mitigation strategies [6,8]. Moreover, the Punisher directly senses resource competition through which synthetic gene expression impairs engineered cells’ growth. Consequently, it is only triggered when synthetic gene expression loss accelerates the mutant cell’s growth, whereas other approaches may exhibit unintended reactions to mutations that do not provide a growth advantage, inadvertently making engineered cell populations less genetically stable (§3.2).

Our design was studied using a coarse-grained resource-aware cell model [5], enabling a holistic view of cell growth’s burden-dependence that underlies mutation spread in cell populations, as well as the contribution of native and synthetic genes to the resource competition sensed by the Punisher, and the effect of antibiotics leveraged to penalize mutations when resistance is disabled by our circuit. Possessing more predictive power than the basic gene expression models that ignore the cellular context, our modelling framework is also less complex than finer-grained cell models. This enables mathematical derivations that elucidate the Punisher’s switching behaviour and allow to determine the threshold value of burden at which it becomes activated. Furthermore, the computational efficiency of cell model simulations allows to define the rates of cells switching between different states in a population based on stochastic single-cell trajectories rather than arbitrary parametrization [7]. Since synthetic genes in our model are described using physiologically relevant gene expression parameters like promoter strength and ribosome affinity, our approach establishes a novel rigorous method of directly linking a circuit’s (burden-dependent) population dynamics to its design parameters.

The Punisher is primarily intended for deployment alongside synthetic genes that hinder cell growth predominantly via gene expression burden, which presently comprise a large proportion of synthetic biology constructs [35]. Our design is also mainly aimed at mutations reducing gene expression wholly or at least significantly, since they cause greater detriment to engineered cell populations’ productivity, spread across populations faster (due to conferring a greater growth advantage to mutant cells), and are thus less likely to be thwarted by clonal interference from functional cells with beneficial native gene mutations [34]. However, the Punisher may still prove useful outside of its intended applications. Namely, it can penalize mutations of metabolically burdensome synthetic genes (electronic supplementary material, Note S2.5). Relatively small reductions in synthetic gene expression—such as mutations of just a few gene copies among many or mutations only partially disabling expression—may still be penalized by the Punisher if its switching threshold $\hat{\Xi }$ is appropriately tuned (electronic supplementary material, Note S2.4). Moreover, even when synthetic gene mutations do not reduce burden below this threshold, the Punisher renders them less favourable than native gene mutations with equivalent growth advantages, contributing to the slowdown of engineered functionality loss by cell populations (electronic supplementary material, Note S4.1).

Although this study includes extensive simulations and analysis of the Punisher’s performance, important avenues of research remain to be explored. While our findings indicate that the Punisher should extend engineered cell populations’ functionality in the face of native gene or partially disabling gene mutations, their population-wide effects could be investigated further by extending our cell population model. However, in this case efficient simulation could prove challenging due to the need to consider more cell states. The Punisher is also yet to be implemented and tested *in vivo*. Nonetheless, past studies provide solid foundations for this effort—indeed, individual features of our design, such as gene self-activation and integrase-mediated disabling of chloramphenicol resistance, have already been achieved and tested in bacteria [7,9].

In conclusion, we have proposed a promising versatile biomolecular controller design, the same genetic implementation of which can improve engineered cell populations’ genetic stability in diverse applications. More generally, the present study represents a showcase and a blueprint for how insights from cell modelling both on a single-cell and population level can facilitate several different aspects of resource-aware biomolecular controller development.

## Data Availability

All data and code used in this publication are available at [21]. Supplementary material is available online [36].
